# Unity Quantum Yield of High‐Entropy Quantum Dots Composited With Photonic Crystals for Information Encryption

**DOI:** 10.1002/advs.75603

**Published:** 2026-05-08

**Authors:** Maoyuan Huang, Ziqiang Tian, Binkun Xie, Haiyang Li, Bo Tan, Chang He, Shiliang Mei, Wanlu Zhang, Changchun Wang, Ruiqian Guo

**Affiliations:** ^1^ Institute for Electric Light Sources College of Intelligent Robotics and Advanced Manufacturing Fudan University Shanghai China; ^2^ State Key Laboratory of Molecular Engineering of Polymers and Department of Macromolecular Science Laboratory of Advanced Materials Fudan University Shanghai China; ^3^ Yiwu Research Institute of Fudan University Yiwu Zhejiang China; ^4^ Zhongshan‐Fudan Joint Innovation Center Zhongshan China

**Keywords:** eco‐friendly, high PLQY, high‐entropy quantum dots, information encryption, photonic crystals

## Abstract

The escalating demand for advanced anti‐counterfeiting and information encryption technologies has driven the exploration of luminescent materials with high quantum yield, multi‐level encoding capability, and long‐term stability. This study extends the high‐entropy paradigm to I‐III‐VI quantum dots (QDs) for the first time, successfully synthesizing CuZnCrGaSe/ZnSe/ZnS (CZCrGSe/ZnSe/ZnS) core/shell/shell high‐entropy QDs via a one‐pot nucleation strategy combined with stepwise hot‐injection shell coating. By optimizing reaction parameters and composition ratios, combined with a precisely designed ZnSe/ZnS double shell for efficient defect passivation, the QDs achieve an emission wavelength of 540 nm and a record‐breaking photoluminescence quantum yield (PLQY) of 100%, which stands as the highest PLQY reported for alloy QDs to date. Integrating these high‐performance QDs into stimulus‐responsive photonic crystals (PCs) yields dual‐mode nanocomposite films capable of exhibiting two reversible optical states: structural color and fluorescent color. The luminescent properties of high‐entropy QDs synergistically modulate with the photonic bandgap of the PCs, enabling multidimensional information encryption. This functionality was validated through a visual encoding/decoding system capable of secure binary code conversion. This work achieves a significant breakthrough in the luminescent performance of alloy QDs and provides a novel strategy for developing eco‐friendly, high‐performance optical encryption materials.

## Graphical abstract

This study first extends the high‐entropy concept to I‐III‐VI quantum dots (QDs) and synthesizes CZCrGSe/ZnSe/ZnS core/shell/shell QDs with a record photoluminescence quantum yield (PLQY) of 100%. Their ZnSe/ZnS double shell efficiently passivates defects and boosts structural stability. Integrating these QDs with stimulus‐responsive photonic crystals realizes dual‐mode optical information encryption.

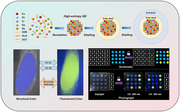

## 1 Introduction

In the contemporary digital era, protecting critical information from forgery and unauthorized access is a major challenge across various industries [1, 2, 3]. Optical encryption technology, leveraging the unique photophysical properties of luminescent materials, has emerged as a powerful solution due to its high security, ease of authentication, and potential for multi‐dimensional encoding [3, 4, 5, 6, 7, 8, 9, 10]. Colloidal quantum dots (QDs) are considered one of the most promising candidates for such applications, thanks to their size‐tunable emission, high photoluminescence quantum yield (PLQY), and solution processability [11, 12, 13, 14, 15, 16, 17, 18, 19, 20, 21, 22, 23, 24, 25, 26]. However, traditional QDs mostly contain toxic elements such as cadmium (Cd) and lead (Pb), which impose inherent toxicity and limit their practical applications [27, 28]. For example, II‐VI QDs contain highly toxic Cd, while most perovskite QDs suffer from environmental degradation sensitivity, compositional instability, and toxicity [27, 28, 29, 30, 31]. As a typical non‐toxic II‐V candidate, InP QDs suffer from high cost and complex synthesis [16, 32]. ZnSe QDs exhibit low toxicity but suffer from limited spectral tunability in the visible region [20, 21]. Among alternatives, I‐III‐VI QDs (e.g., Cu‐In‐S, Cu‐In‐Se, Ag‐Ga‐S, Cu‐Ga‐S, Cu‐Ga‐Se) have been recognized as important candidates due to their non‐toxicity, widely tunable emission wavelengths, and facile synthesis [33, 34, 35, 36, 37, 38, 39, 40, 41, 42, 43, 44, 45, 46, 47].

High‐entropy alloys consist of at least five elements with approximately equal atomic ratios. They have garnered extensive attention due to their unique properties, including high‐entropy effect, lattice distortion effect, sluggish diffusion, and “cocktail effect” [48, 49, 50]. In recent years, the concept of high entropy has been widely applied in various functional materials. The recent emergence of high‐entropy functional materials provides a breakthrough paradigm for designing eco‐friendly QDs with exceptional performance [51, 52, 53]. As demonstrated in halide perovskite systems, the introduction of multiple metal cations at a single lattice site significantly enhances configurational entropy, which thermodynamically stabilizes the crystal structure and kinetically suppresses ion migration [54, 55]. This “high‐entropy effect” endows materials with extraordinary tolerance to heat, light, and solvents, addressing the long‐standing stability issues of perovskite QDs. Inspired by this, we envision extending the high‐entropy concept to I‐III‐VI QDs, promising a new class of eco‐friendly luminescent materials with high PLQY.

Parallel to composition engineering, precise core/shell heterostructure design is crucial for achieving high PLQY. Growing a wider bandgap shell on the QD core is a reliable strategy to passivate surface traps and suppress non‐radiative recombination. For instance, Liu et al. demonstrated that constructing a rigid ZnS shell on CuInS_2_ QDs yielded an excellent PLQY of 92.1% by inhibiting cation exchange and Auger recombination [44]. In 2024, Lu et al. achieved a record‐breaking PLQY of 96.4% for AgGaS QDs via a ZnS/ZnS double‐shell strategy, which effectively reduced lattice mismatch and minimized interface defects [45]. These works highlight the critical role of precise shell structures in maximizing luminescent efficiency. Applying this principle, a carefully designed ZnSe/ZnS double shell on the high‐entropy CuZnCrGaSe (CZCrGSe) core is expected to provide optimal surface coverage and confinement effects, thereby pushing its emission performance close to the theoretical limit.

To go beyond the functionalities of standalone luminescent materials, their integration into photonic crystals (PCs) offers a powerful approach to create intelligent encryption systems [8]. PCs are able to manipulate light propagation in unique ways due to their periodic dielectric structures and photonic band gaps [56, 57]. By embedding QDs into a stimulus‐responsive PC matrix, the resulting composites not only exhibit bright and stable emission but also enable dynamic emission regulation by the photonic bandgap in response to external stimuli, introducing an additional, highly secure dimension to encryption codes.

Herein, this study for the first time extends the high‐entropy concept to the I‐III‐VI QD system, proposing a hot injection method to synthesize high‐luminescence CZCrGSe/ZnSe/ZnS QDs. The synthesized CZCrGSe/ZnS/ZnS QDs exhibit an exceptional PLQY of 100%, representing the highest PLQY reported to date for alloy QDs. The high‐entropy alloy core is formed via a one‐pot method, while the outer ZnSe and ZnS shells are prepared by stepwise injection of zinc precursors. The formation of the inner ZnSe shell reduces the lattice mismatch between the QD core and the outer ZnS shell, facilitating the growth of a thicker outer shell. Furthermore, the effects of nucleation temperature and cation/anion ratio on the luminescent properties of high‐entropy QDs are investigated. Subsequently, the high‐performance high‐entropy QDs are integrated into PCs for advanced information encryption. After assembling QDs within the PCs, the nanocomposite exhibits synergistic effects: the fabricated dual‐mode PC film demonstrates two distinct optical states—structural color and fluorescent color. Leveraging its exceptional optical properties, this dual‐mode PC film has been applied in visual encoding and decoding systems. This work establishes a new blueprint for developing high‐performance, secure, and durable optical encryption materials.

## 2 Results and Discussion

**FIGURE 1:**
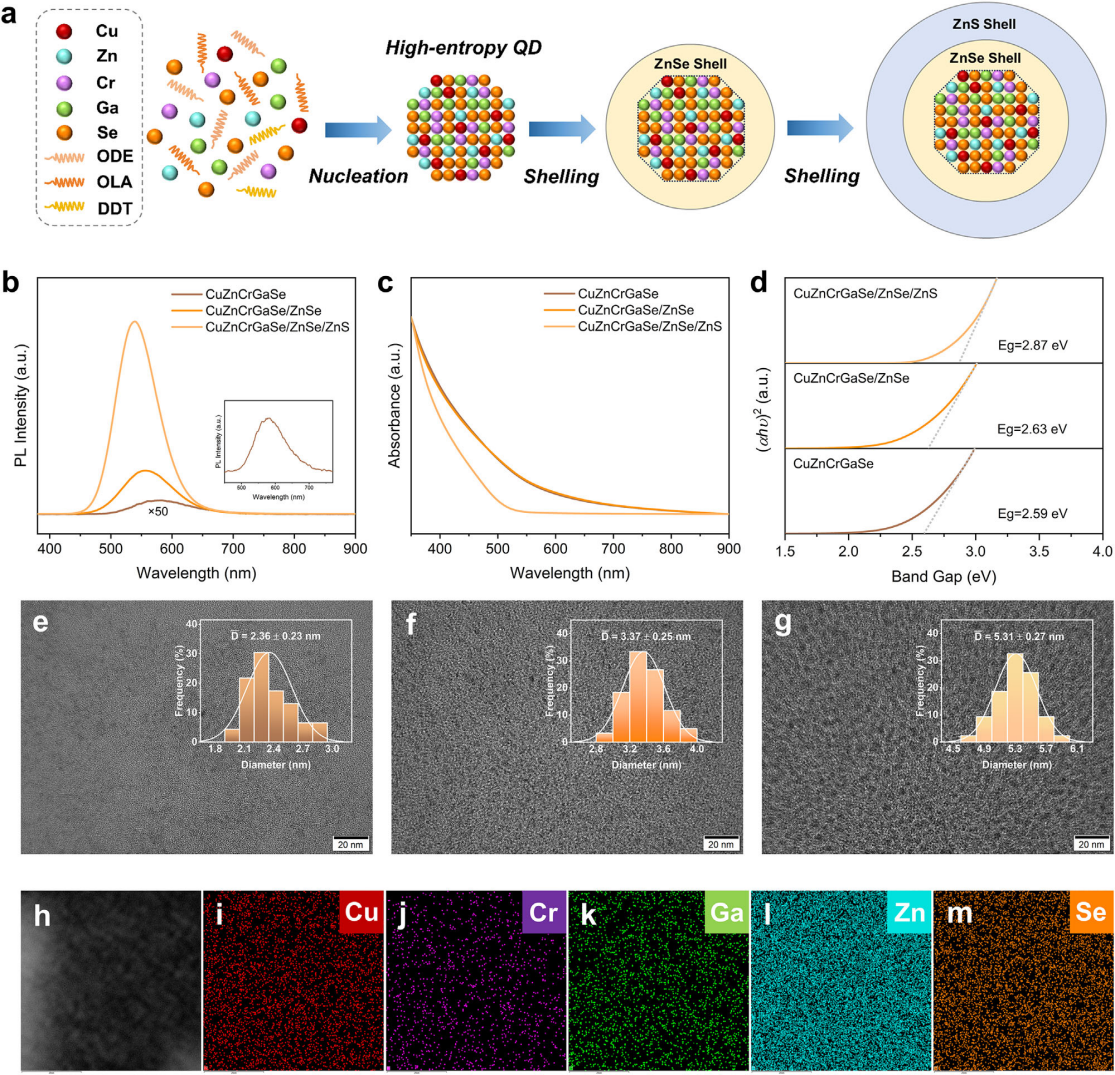
(a) Schematic diagram of CZCrGSe/ZnSe/ZnS growth process. (b) PL spectra (*λ*_ex_ = 365 nm), (c) absorption spectra, and (d) Tauc plots of CZCrGSe QDs, CZCrGSe/ZnSe QDs, and CZCrGSe/ZnSe/ZnS QDs. TEM images of (e) CZCrGSe QDs, (f) CZCrGSe/ZnSe QDs, and (g) CZCrGSe/ZnSe/ZnS QDs. Insets show the corresponding size distribution histograms. (h) HAADF‐STEM image of CZCrGSe/ZnSe/ZnS QDs. (i–m) The corresponding elemental mapping images for Cu, Zn, Cr, Ga, and S elements of CZCrGSe/ZnSe/ZnS QDs.

Eco‐friendly high‐entropy QDs were first synthesized via a one‐pot nucleation method combined with two‐step hot‐injection shell coating. Copper iodide (CuI), zinc iodide (ZnI_2_), chromium(III) acetylacetonate (Cr(acac)_3_), gallium acetylacetonate (Ga(acac)_3_), selenium powder (Se), oleylamine (OLA), 1‐octadecene (ODE), and 1‐dodecanethiol (DDT) were added to a three‐necked flask and heated for nucleation to prepare CZCrGSe core QDs. Subsequently, the ZnI_2_ precursor solution was slowly injected four times to react with excess Se in the system to form a ZnSe shell, yielding CZCrGSe/ZnSe QDs. Then, the ZnS shell solution was slowly added, finally forming CZCrGSe/ZnSe/ZnS QDs. Figure 1a shows a schematic diagram of the growth process of high‐entropy CZCrGSe/ZnSe/ZnS QDs, and a schematic of the QD preparation route is presented in Figure S1. It is generally accepted that radiative transitions caused by donor‐acceptor pair recombination are the main source of luminescence in I‐III‐VI QDs. The formation of ZnSe and ZnS shells can significantly enhance their luminescent properties. In this study, CZCrGSe core QDs, CZCrGSe/ZnSe QDs, and CZCrGSe/ZnSe/ZnS core/shell QDs were synthesized as described above. Figure 1b displays the photoluminescence (PL) spectra of CZCrGSe QDs, CZCrGSe/ZnSe QDs, and CZCrGSe/ZnSe/ZnS QDs. After ZnSe shell coating, the emission wavelength blue‐shifted from 581 nm to 558 nm, the PL intensity increased, and the PLQY improved from 5% to 43%. The blue shift of the emission peak can be attributed to ion exchange caused by the diffusion of Zn ions into the high‐entropy core, and this partial alloying further leads to a slight expansion of the bandgap. After further coating with the ZnS shell, both the PL and absorption spectra exhibited significant blue shifts, with the emission wavelength blue‐shifting from 558 to 540 nm (Figure 1b,c). For the absorption spectra, no obvious exciton peaks were observed, which is consistent with the general characteristics of various I‐III‐VI QDs [35, 41]. The Tauc plot derived from the absorption spectra showed that the bandgap increased from 2.63 to 2.87 eV (Figure 1d). Meanwhile, the PL intensity was greatly enhanced, and the PLQY surprisingly increased to 100% (Figure S2). To the best of our knowledge, this is the highest PLQY reported to date among all I‐III‐VI QDs (Table S1). To investigate the reasons for the PL enhancement and spectral changes, morphological, structural, and elemental characterizations of the QDs were performed.

Information on the microstructure and particle size distribution of CZCrGSe QDs, CZCrGSe/ZnSe QDs, and CZCrGSe/ZnSe/ZnS QDs is shown in the transmission electron microscopy (TEM) results of Figure 1e–g and Figure S3. The TEM results in Figure S3 show that the high‐entropy QDs exhibit clear circular features in the 2D plane, and shell coating does not change the morphology of the QDs. As observed in Figure S3a, the CZCrGSe core QDs exhibited obvious aggregation. Aggregated QDs are prone to energy transfer between particles due to reduced interparticle spacing, leading to decreased luminescent performance. The QDs after shell coating showed good dispersibility (Figure S3b,c). As shown in Figure 1e–g, the particle size distribution histograms obtained via size measurement indicate that the QDs all have relatively uniform size distributions. The sizes of CZCrGSe QDs, CZCrGSe/ZnSe QDs, and CZCrGSe/ZnSe/ZnS QDs are 2.36, 3.37, and 5.31 nm, respectively, and the size uniformity is improved with shell coating. The significant increase in the size of high‐entropy QDs after the second coating is due to the formation of a thicker ZnS shell. As shown in Figure S4a, the high‐resolution TEM (HRTEM) image of CZCrGSe QDs displays continuous lattice fringes with an interplanar spacing of 0.32 nm, which can be attributed to the (112) plane of the chalcopyrite phase. The interplanar spacings of the QDs in Figure S4b,c are 0.33 and 0.31 nm, corresponding to the (111) plane lattice spacings of zinc blende ZnSe and ZnS, respectively, indicating the successful coating of ZnSe and ZnS shells on the high‐entropy CZCrGSe core. In addition, high‐angle annular dark‐field scanning transmission electron microscopy (HAADF‐STEM) and energy dispersive spectroscopy (EDS) mapping confirmed the uniform distribution of all constituent elements (Cu, Cr, Ga, Zn, and Se) in the lattices of CZCrGSe QDs, CZCrGSe/ZnSe QDs, and CZCrGSe/ZnSe/ZnS QDs (Figures S5 and S6; Figure 1h–m). The elemental content of the high‐entropy QDs was further analyzed via energy dispersive spectroscopy (EDS), as shown in Figure S7. After ZnSe shell coating, the Zn content significantly increased from 10.29% (core‐only QDs) to 39.00%, and further increased to 59.81% after ZnS shell coating. The results indicate that a large amount of Zn remains on the outer layer of the QDs to passivate the surface defects of the high‐entropy core.

**FIGURE 2:**
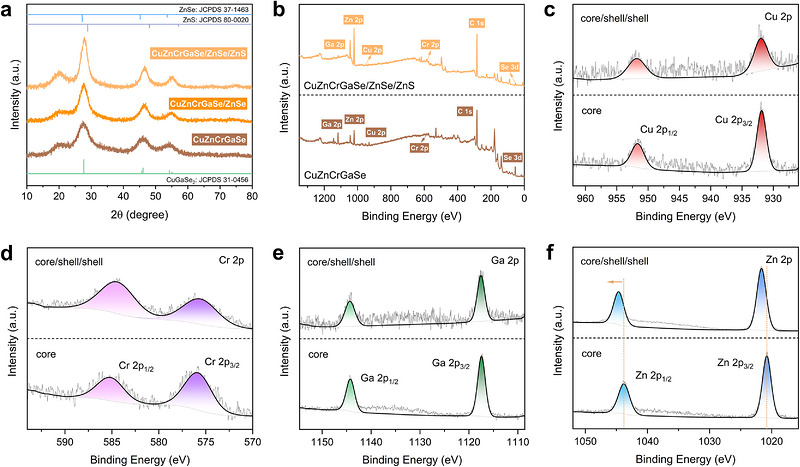
(a) XRD patterns of CZCrGSe QDs, CZCrGSe/ZnSe QDs, and CZCrGSe/ZnSe/ZnS QDs. (b) Survey scan XPS spectra of CZCrGSe QDs and CZCrGSe/ZnSe/ZnS QDs. High‐resolution scan XPS spectra of (c) Cu 2p, (d) Cr 2p, (e) Ga 2p, and (f) Zn 2p for CZCrGSe QDs and CZCrGSe/ZnSe/ZnS QDs.

The crystal structure of the QDs was characterized via X‐ray diffraction (XRD) measurements, and the results are shown in Figure 2a. The XRD pattern of CZCrGSe QDs was calibrated using Jade software. The results showed distinct diffraction peaks at 2θ diffraction angles of 27.7°, 46.1°, and 54.2°, which are consistent with the standard card of tetragonal chalcopyrite CuGaSe_2_ (JCPDS 31–0456). The 2θ diffraction angles correspond to the (112), (220)/(204), and (312)/(116) planes, respectively. After coating the CZCrGSe core with the ZnSe shell, the position of the diffraction peak shifted to a higher angle, indicating a decrease in the crystal lattice constant. This is due to the lattice strain between the ZnSe shell and the CZCrGSe core during the epitaxial growth of the ZnSe shell on the surface of the CZCrGSe core. The CZCrGSe core is under compressive strain, and the ZnSe shell is under tensile strain. The continuous growth of the ZnS shell caused the diffraction peak to shift to a higher angle, gradually approaching the ZnS phase, which further confirms the formation of the core/shell/shell structure.

To further investigate the composition and elemental valence state information of the high‐entropy QDs, XPS characterization was performed on CZCrGSe core QDs and CZCrGSe/ZnSe/ZnS QDs. As shown in Figure 2b, characteristic peaks of Cu 2p, Zn 2p, Cr 2p, Ga 2p, and Se 3d can be observed in the XPS survey spectra. The high‐resolution XPS spectra of each element were further analyzed, as shown in Figure 2c–f. The binding energies of Cu 2p for CZCrGSe QDs are 951.63 and 931.87 eV, corresponding to the Cu 2p_1/2_ and Cu 2p_3/2_ orbitals, indicating that Cu in the QDs is +1 [43, 44]. Similarly, the binding energies of Zn 2p are 1043.77 and 1020.78 eV, the binding energies of Cr 2p are 585.15 eV (note: original text may have a typo, corrected for consistency), and the binding energies of Ga 2p are 1144.23 and 1117.44 eV, which are basically consistent with previous literature reports, indicating that the valences of Zn, Cr, and Ga in the QDs are +2, +3, and +3, respectively. After the growth of the ZnS shell, there were no obvious changes in the binding energy positions of the Cu 2p and Ga 3d characteristic peaks (Figure 2c–e), indicating that the coordination environment of the Cu, Cr, and Ga lattice sites in the CZCrGSe core crystal changed minimally during the growth of the ZnS shell, confirming the formation of the CZCrGSe/ZnSe/ZnS core‐shell structure rather than an alloyed structure. As shown in Figure 2f, compared with the initial CZCrGSe core QDs, the Zn 2p characteristic peak shifted to a higher binding energy by approximately 0.85 eV, indicating an increase in the electron density around Zn atoms, which is consistent with the passivation effect of the ZnS shell on the surface of high‐entropy QDs.

**FIGURE 3:**
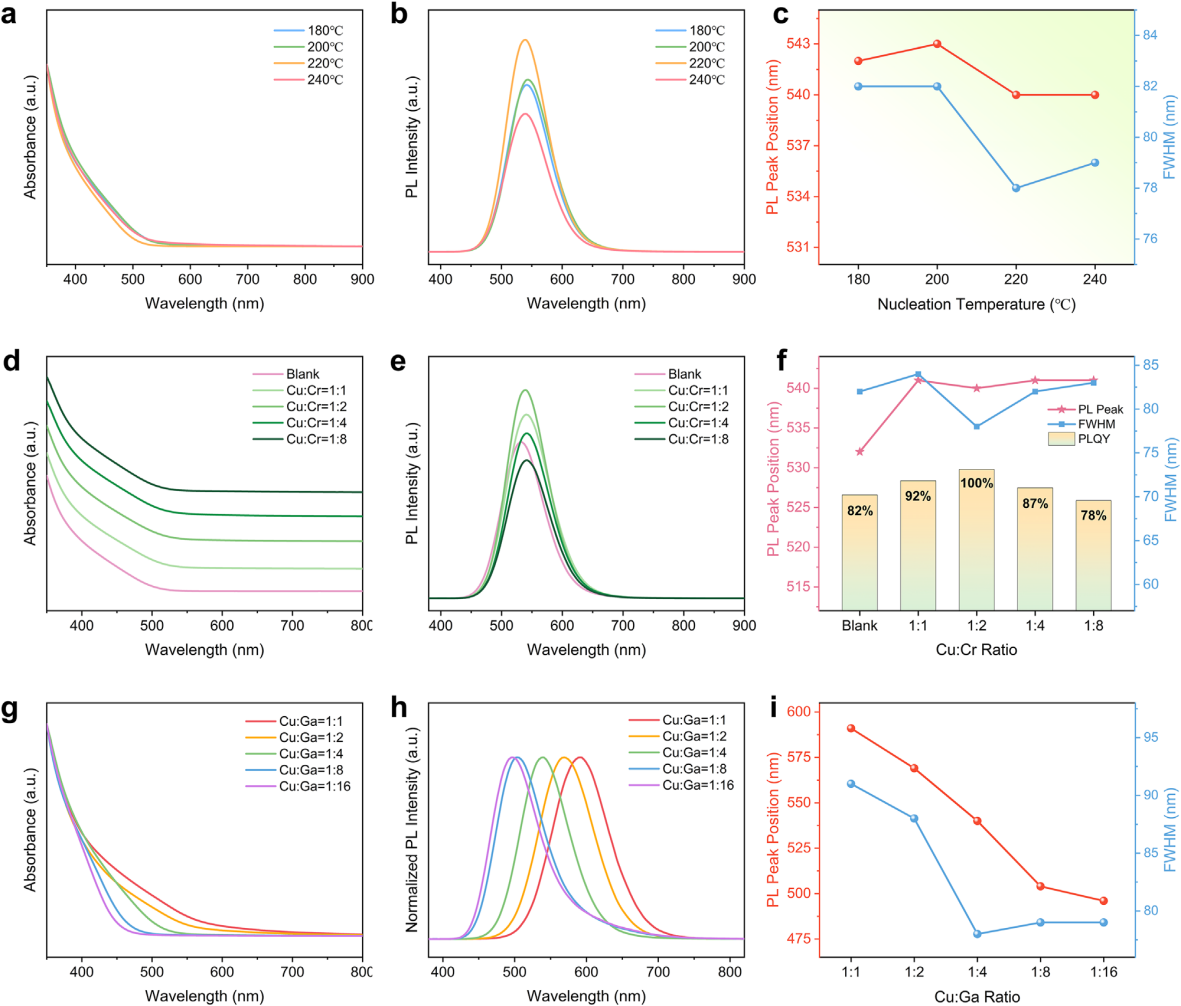
(a) Absorption spectra, (b) PL spectra, and (c) PL peak positions (left axis) and FWHM (right axis) of CZCrGSe/ZnSe/ZnS QDs synthesized with different nucleation temperatures. (d) Absorption spectra, (e) PL spectra, and (f) PL peak positions, FWHM, and PLQY of CZCrGSe/ZnSe/ZnS QDs synthesized with different Cu:Cr ratios. (g) Absorption spectra, (h) normalized PL spectra, and (i) PL peak positions and FWHM of CZCrGSe/ZnSe/ZnS QDs synthesized with different Cu:Ga ratios.

To achieve high PL performance of CZCrGSe/ZnSe/ZnS QDs, optimizing reaction parameters is crucial. The effect of nucleation temperature on the optical properties of QDs was systematically studied. Absorption and PL spectra of CZCrGSe/ZnSe/ZnS QDs synthesized at different nucleation temperatures are shown in Figure 3a,b. When the nucleation temperature increased from 180°C to 220°C, the absorption spectrum showed no obvious change, while the PL intensity increased significantly, and the full width at half maximum (FWHM) narrowed from 82 to 78 nm (Figure 3c). When the nucleation temperature further increased to 240°C, the PL intensity decreased obviously. These observations suggest that an appropriate nucleation temperature improves the uniformity of emissive states and reduces defect‐related broadening, while excessively high temperature may induce additional disorder or defect formation during nucleation and growth. The PLE spectrum showed that the optimal excitation wavelength of the high‐entropy QDs is 399 nm (Figure S8). We further explored the effect of intermediate shell coating time on luminescence. Figure S9a–c shows the absorption and PL spectra of CZCrGSe/ZnSe/ZnS QDs after stepwise injection of the ZnI_2_ precursor solution. Obviously, after the first few injections, the ZnSe shell significantly enhanced the PL intensity of the almost non‐luminescent CZCrGSe core, while more injection batches showed limited enhancement. After the fourth injection of the ZnI_2_ precursor solution (ZnSe shell coating for 40 min), the PL intensity reached the maximum value. With the reduction of non‐radiative recombination, the PL intensity was significantly enhanced, while the absorption spectrum remained almost unchanged. The peak position shifted from 573 to 558 nm, which may be due to the penetration of Zn ions into the core, leading to cation exchange and a slight expansion of the bandgap.

Due to the high tolerance of alloy QDs to non‐stoichiometric composition, spectral regulation by adjusting the ratio of various elements is a common method. We studied the effect of cation composition changes on the spectral regulation of CZCrGSe/ZnSe/ZnS QDs. Fixing the amount of Cu precursor and changing the content of Cr precursor, five groups of samples with different Cu:Cr feeding ratios were obtained. Absorption and PL spectra of QDs with different Cr contents are shown in Figure 3d,e. The PL intensity of QDs first increased and then decreased with the increase of Cr content, and reached the maximum when the Cu:Cr feeding ratio was 1:2 (Figure 3e). The introduction of Cr element caused a slight red shift of the emission peak position of QDs, and the wavelength of Cr‐containing high‐entropy QDs was stable around 540 nm (Figure 3f). The PLQY was measured at an optimal excitation wavelength of 399 nm. It was found that the PLQY increased from 82% to 100% as the Cu:Cr feeding ratio changed from 1:0 to 1:2, and the FWHM was 78 nm. When the Cu:Cr feeding ratio decreased to 1:8, the PL intensity significantly decreased from 100% to 78%, and the FWHM increased to 83 nm. Higher Cr content would introduce electron traps in the conduction band, which recombine with holes, leading to increased non‐radiative recombination, thereby increasing the FWHM and decreasing the PLQY.

Fixing the Cu:Cr feeding ratio at 1:2 and changing the content of Ga precursor, five groups of samples with Cu:Ga feeding ratios of 1:1, 1:2, 1:4, 1:8, and 1:16 were obtained. As the Cu:Ga molar ratio decreased from 1:1 to 1:16, the emission peak gradually blue‐shifted from 590 to 496 nm, and the absorption spectrum also showed a consistent blue shift (Figure 3g–i). This may be attributed to the weakening repulsive force between the Cu d and Se p orbitals as the Cu content decreases, leading to a reduction in the maximum valence band energy and consequently broadening the bandgap [36]. This behavior is analogous to that observed in CuInS_2_ QDs [58]. The Cu:Ga ratio also has a significant effect on the PL intensity of high‐entropy QDs. It is generally believed that the PL of I‐III‐VI QDs originates from carrier recombination in defect states within the bandgap, and appropriate Cu defects are beneficial to enhance PL intensity. The PL intensity reached the maximum when the Cu:Ga ratio was 1:4 (Figure S10). When the Ga:Cu ratio was 1:1, the Cu vacancies in the QDs were insufficient, and the emission centers were few, so the emission of QDs was weak. When the Cu content is too low, the crystal structure of QDs is compromised, leading to the formation of numerous defects. This results in increased non‐radiative recombination and a decrease in the fluorescence intensity of the QDs.

**FIGURE 4:**
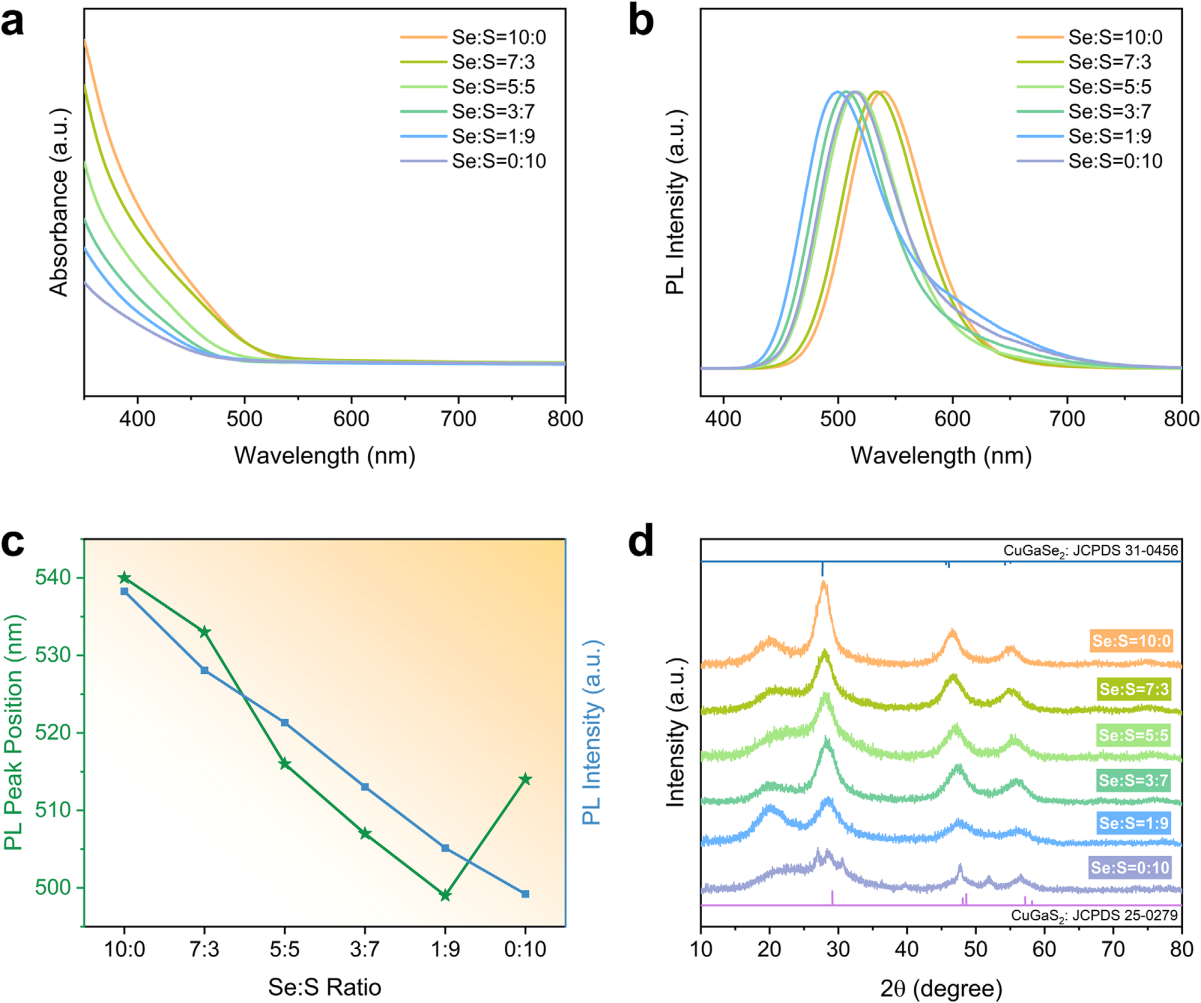
(a) Absorption spectra, (b) normalized PL spectra, (c) PL peak positions (left axis) and PL intensity (right axis), and (d) XRD patterns of CZCrGSe_x_S_1‐x_/ZnSeS/ZnS QDs synthesized with different Se:S ratios.

From the above studies, it was found that the strongest PL was achieved when the nucleation temperature was 220°C, the Cu:Cr feeding ratio was 1:2, and the Cu:Ga feeding ratio was 1:4. We further synthesized CZCrGSe_x_S_1‐x_/ZnSeS/ZnS QDs with Se:S precursor molar ratios of 10:0, 7:3, 5:5, 3:7, 1:9, and 0:10 by anion high‐entropy modification, fixing the Cu:Zn:Cr:Ga precursor molar ratio at 1:4:2:4 and the total amount of Se and S precursors at 1.3 mmol. As shown in Figure 4a,b, as the Se:S ratio decreased from 10:0 to 1:9, the absorption and spectra also showed a consistent blue shift, and the emission wavelength could be tuned from 540 to 499 nm. We attribute this to the gradual increase in the content of wide‐bandgap Ga_2_S_3_ component in the alloy QDs with the increase of S content, and the bandgap of the alloy increases accordingly, leading to the blue shift of the wavelength [40]. With the increase of S dosage, the PL intensity also decreased, and the luminescent color change from yellowish green to cyan can be seen in the inset of Figure S11 (Figure 4c; Figure S11). Notably, the emission wavelength of CZCrGS/ZnS/ZnS QDs synthesized with a Se:S precursor molar ratio of 0:10 red‐shifted to 514 nm. We speculate that the lattice structure of the high‐entropy core was disordered to a certain extent at this time. For the S system, since only the ZnS shell can be formed, there is a larger lattice mismatch between the core and the shell, and the spectrum also has an obvious tailing phenomenon in the long wavelength region [36, 40]. The corresponding XRD patterns in Figure 4d show that all the prepared CZCrGSe_x_S_1‐x_/ZnSeS/ZnS QDs have three distinct diffraction peaks of the tetragonal chalcopyrite structure. With the increase of S content, the diffraction peak position shifted from the CuGaSe_2_ direction to the higher angle direction of CuGaS_2_, confirming the successful formation of the CZCrGSe_x_S_1‐x_/ZnSeS/ZnS structure. In addition, when the Se:S feeding ratio was 0:10, impurity peaks appeared in the XRD pattern of the sample, which is consistent with our previous speculation of lattice disorder.

After investigating the synthesis conditions and cation/anion ratio of CZCrGSe_x_S_1‐x_ /ZnSeS/ZnS QDs, we fixed the nucleation temperature at 220°C, the Cu:Cr molar ratio at 1:2, the Cu:Ga molar ratio at 1:4, and the Se:S molar ratio at 5:5, and introduced the Mn element to conduct experimental research on seven‐component high‐entropy CZMnCrGSeS/ZnSeS/ZnS QDs. Benefiting from the high‐entropy lattice with high configurational entropy and lattice distortion, the host structure provides a uniform and stable coordination environment for Mn^2+^ doping, which helps suppress ion segregation and phase separation, thus enabling homogeneous doping and reliable luminescence.

**FIGURE 5:**
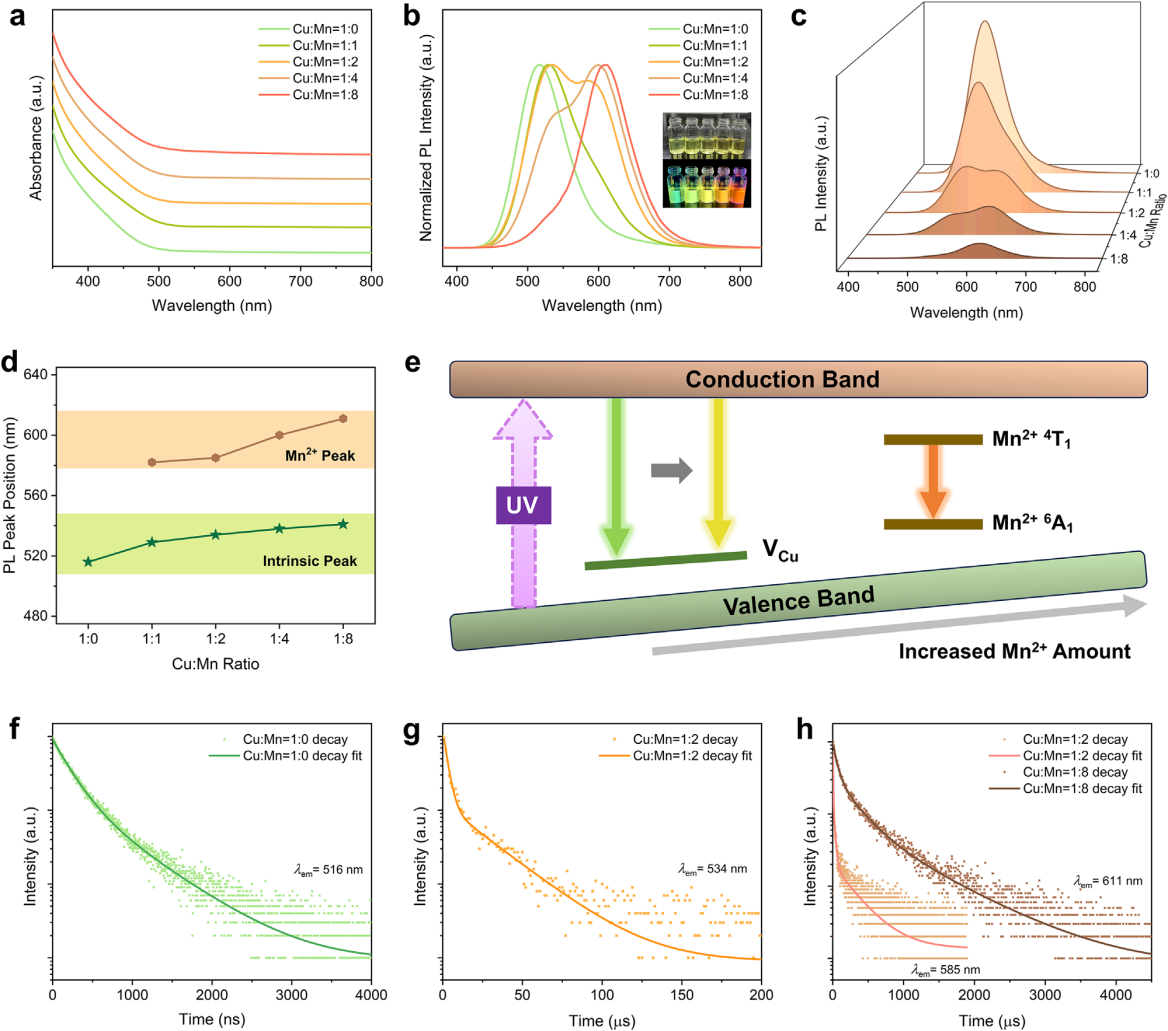
(a) Absorption spectra, (b) normalized PL spectra, and (c) 3D PL spectra of CZMnCrGSeS/ZnSeS/ZnS QDs synthesized with different Cu:Mn ratios. (d) PL peak position of intrinsic emission and Mn‐related emission with different Cu:Mn ratios. (e) Schematic illustration of the radiative pathways in CZMnCrGSeS/ZnSeS/ZnS QDs. TRPL and fitting curves of CZMnCrGSeS/ZnSeS/ZnS QDs synthesized with (f) Cu:Mn = 1:0 and (g) Cu:Mn = 1:2 measured at the intrinsic peak. (h) TRPL and fitting curves of CZMnCrGSeS/ZnSeS/ZnS QDs synthesized with Cu:Mn = 1:2 and Cu:Mn = 1:8 measured at the Mn‐related peak.

With the introduction of Mn, the absorption spectrum of QDs showed no obvious change (Figure 5a). As shown in Figure 5b, the introduction of Mn caused dual‐peak emission of high‐entropy QDs. The peak wavelength around 530 nm is the intrinsic emission of high‐entropy QDs, and the peak wavelength around 595 nm is the Mn^2+^ energy level‐related composite luminescence. With the increase of the Mn/Cu ratio, the intrinsic emission intensity gradually decreased, accompanied by an increase in the relative emission intensity of Mn^2+^, indicating a competitive emission mechanism between the two (Figure 5c). This is because high concentrations of Mn^2+^ may produce more non‐radiative energy transfer with the defect levels of the host, leading to a decrease in PL intensity. The change in visual fluorescence is shown in the inset of Figure 5b. With the increase of Mn content, the PL gradually changed from green to orange‐red. Figure 5d shows the changes in the intrinsic peak and Mn^2+^ emission peak wavelengths of CZMnCrGSeS/ZnSeS/ZnS QDs with Mn content. With the increase of Mn content, the intrinsic emission wavelength of QDs shows a stable red shift trend. As shown in Figure 5e, for CZMnCrGSeS/ZnSeS/ZnS QDs, their intrinsic emission mainly comes from the radiative recombination between the conduction band and the defect states caused by Cu vacancies, so the position of their emission peak is mainly affected by the bandgap of QDs. The emission in the orange light band comes from the d‐d transition of Mn from the first excited state ^4^T_1_ to the ground state ^6^A_1,_ so the position of its emission peak is usually not affected by the bandgap and doping concentration of the host QDs. However, in Figure 5d, with the increase of Mn concentration, the Mn^2+^ emission shows a slight red shift. This shift can be explained by the slight modulation of the band structure and defect distribution caused by Mn^2+^ doping, which narrows the effective optical bandgap of the host. For Mn^2+^ emission, the peak position is normally dominated by the ligand field and less affected by the host bandgap. However, a slight red shift is observed at high doping levels, which is attributed to Mn‐Mn interactions. At high concentrations, the average distance between Mn^2+^ ions decreases, leading to exchange coupling between adjacent Mn^2+^ centers and slight splitting of the excited levels, resulting in a red‐shifted emission [59, 60, 61].

We monitored the time‐resolved PL (TRPL) decay curves of the two emission components (Figure 5f‐h). The measured fluorescence decay curves can be fitted by the three‐exponential function (Equation 1):

(1) $$I\left(t\right)=A_{1}e^{-t/\tau_{1}}+A_{2}e^{-t/\tau_{2}}+A_{3}e^{-t/\tau_{3}}$$

where *τ*_1_, *τ*_2_, and *τ*_3_ represent the decay times of fluorescence emission, and *A*_1_, *A*_2_, and *A*_3_ represent the proportions of the corresponding fluorescence lifetimes. The average fluorescence lifetime is calculated by Equation (2):

(2) $$\tau_{\mathrm{ave}}=\frac{\sum A_{i}\tau_{i}^{2}}{\sum A_{i}\tau_{i}}$$

The PL decay profile consists of three distinct decay components: a fast‐decaying component, a second component, and a third component [39, 47, 62]. Specifically, the fast‐decaying component originates from non‐radiative recombination induced by surface defect states, while the second and third components correspond to band‐hole recombination arising from internal defect states and donor‐acceptor pair (DAP) recombination, respectively. For the high‐entropy QDs without Mn, the average fluorescence lifetime of their intrinsic emission was determined to be 461.23 ns (Table S2). After Mn doping, the average lifetime of intrinsic emission is prolonged, accompanied by the typical millisecond‐scale lifetime of Mn^2+^ emission. This phenomenon reveals that energy transfer preferentially consumes short‐lived excited states, increasing the relative contribution of long‐lived components and thus prolonging the apparent average lifetime. The QDs with a Cu:Mn feeding ratio of 1:2 exhibited a remarkable increase in the average fluorescence lifetime of intrinsic emission, reaching 24 µs, alongside an average fluorescence lifetime of 0.31 ms for Mn^2+^‐related emission. As the Mn content in the QDs further increased, the average fluorescence lifetime of Mn^2+^ emission for the QDs with a Cu:Mn feeding ratio of 1:8 escalated to 0.67 ms (Table S3). These results are consistent with the lifetime range of Mn‐doped emissions reported in the literature, which further validates the origins of the two emission components [24, 61, 63, 64].

Stability is a critical index for the practical applications of luminescent nanomaterials. In this work, the ambient stability, thermal stability, and UV irradiation stability of the optimized CZCrGSe/ZnSe/ZnS QDs were systematically investigated. In the ambient stability test, the colloidal dispersion of QDs was stored for 240 h under room temperature, ambient air, and natural light (Figure S12). The fluorescence intensity remained 93.4% of the initial value with no obvious shift in the emission peak, demonstrating excellent long‐term ambient stability. The thermal stability test shows that the PL intensity of the QDs still stays above 90% of the initial value after annealing at 80°C for 120 h. For UV stability, the PL intensity of the QDs decreases only slightly after continuous irradiation with 365 nm UV light for 12 h, and still retains more than 75% of the initial level, exhibiting outstanding photobleaching resistance. Such excellent stability stems from the efficient defect passivation effect of the ZnSe/ZnS double‐shell structure and the lattice stabilization effect endowed by the high‐entropy composition, which guarantees the long‐term stable application of the QDs in optical encryption.

**FIGURE 6:**
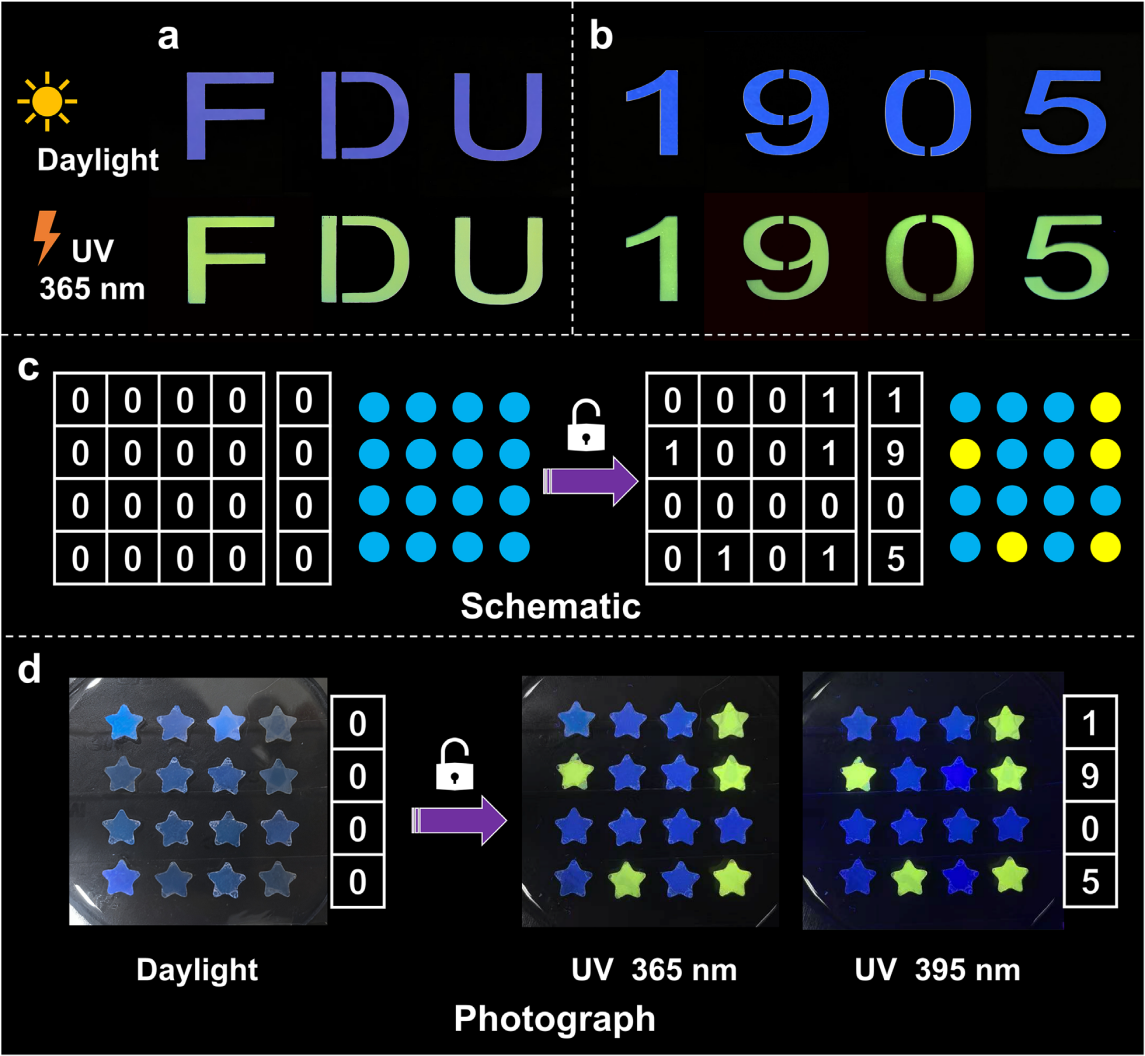
Photos of the pattern (a) “FDU” and (b) “1905” under daylight and UV irradiation (*λ* = 365 nm), respectively. (c) Schematic of the secondary encoding models of “1905”. (d) Photos of the secondary encoding models of “1905”.

By co‐assembling PS@P(EA‐co‐AA) core‐shell nanoparticles with CZCrGSe/ZnSe/ZnS QDs with a PLQY of 100% into a highly ordered periodic photonic structure, dual‐mode high‐entropy QD composited PC (HE‐QD@PC) films were fabricated. The prepared dual‐mode HE‐QD@PC films have two different optical states, namely structural color mode and fluorescent color mode. As shown in Figure S13, the dual‐mode HE‐QD@PC films display two reversible optical responses. Under sunlight irradiation, the ordered photonic‐crystal structure gives rise to structural color through selective reflection of visible light. Under UV excitation, the embedded high‐entropy QDs generate bright fluorescence. The switching between these two optical modes is reversible. As shown in Figure 6a, the letters “FDU” were cut from a dual‐mode HE‐QD@PC film, showing a bright blue structural color under daylight with high saturation and good monochromaticity, which originates from the large number of highly ordered photonic microcrystals in the film. Under 365 nm UV light excitation, the dual‐mode HE‐QD@PC film produces PL due to UV excitation, showing a bright yellowish green color. Figure 6b shows the structural color mode and fluorescent color mode of the number “1905” under daylight and 365 nm UV light excitation, respectively. Due to the two different optical states of the dual‐mode film under different light sources and the reversibility of switching, multi‐level information encryption can be achieved, ensuring its applicability in information storage and multi‐level anti‐counterfeiting without complex preparation processes.

As a proof of concept, we combined dual‐mode HE‐QD@PC films with QD‐free PC films for information encryption via visual encoding and decoding. We designed an information encryption system based on binary codes (Figure 6c). We stipulated that if a point shows blue, it is recorded as “0”; if it shows yellowish green, it is recorded as “1”. As shown in Figure 6d, for the information encryption system, first, dual‐mode PC films with different structural colors or fluorescent colors and traditional PC films were cut into pentagram shapes of the same shape and size. Then, 16 pentagram patterns were selected from the dual‐mode HE‐QD@PC films and traditional PC films and arranged into a 4 × 4 array in a specific combination. Finally, the array was encapsulated into a polydimethylsiloxane (PDMS) matrix to form the carrier of the visual encoding‐decoding system. Both showed the structural color blue of PCs under daylight, and converting the binary code of each column into a decimal number yielded the encrypted invalid information “0000”. Under UV light excitation, the pentagrams cut from the dual‐mode HE‐QD@PC films showed yellowish green, and converting the binary code of each column into a decimal number decoded the valid information “1905”. This demonstrates its broad application potential in optical anti‐counterfeiting and information encryption.

## 3 Conclusion

In conclusion, this study attains multiple pivotal breakthroughs in the design, synthesis, and application of high‐entropy luminescent materials for information encryption. We successfully extended the high‐entropy concept to the I‐III‐VI QD system for the first time, and synthesized CZCrGSe/ZnSe/ZnS high‐entropy QDs with a record PLQY of 100%. This PLQY value is the highest reported to date for I‐III‐VI QDs. The luminescent performance was maximized by optimizing key synthesis parameters, including a nucleation temperature of 220°C, a Cu:Cr molar ratio of 1:2, and a Cu:Ga molar ratio of 1:4. The ZnSe/ZnS double‐shell structure effectively mitigates lattice mismatch and passivates surface defects, ensuring the material possesses both superior luminous efficiency and robust structural stability. The CZCrGSe/ZnSe/ZnS QDs demonstrate excellent stability under ambient conditions, together with favorable thermal stability and UV stability. Furthermore, anion regulation (tuning the Se:S ratio) achieves tunable emission wavelengths ranging from 499 to 540 nm, highlighting the flexibility of the high‐entropy design strategy. The introduction of Mn into the high‐entropy system yields CZMnCrGSeS/ZnSeS/ZnS QDs with dual‐peak emission (i.e., intrinsic emission and Mn^2+^‐associated emission), broadening the material's functional versatility. Additionally, integrating high‐entropy QDs with PS@P(EA‐co‐AA) core‐shell nanoparticles via molecule‐mediated shear‐induced assembly (MSAT) produces dual‐mode PC films. These films enable reversible switching between structural color under daylight and fluorescent color upon UV excitation, offering an additional multi‐level encoding dimension for information encryption. A binary code‐based proof‐of‐concept encryption system realizes secure information storage and decoding, validating the practical potential for advanced anti‐counterfeiting. Overall, this work surmounts the PLQY performance bottleneck of conventional I‐III‐VI QDs and establishes a novel paradigm for integrating high‐entropy materials with PCs for smart encryption. The inherent high‐entropy characteristics and exceptional optical properties of these eco‐friendly QDs offer new insights for the functional applications of QDs.

## Conflicts of Interest

The authors declare no conflicts of interest.

## Supporting information




**Supporting File**: advs75603‐sup‐0001‐SuppMat.docx.

## Data Availability

The data that support the findings of this study are available from the corresponding author upon reasonable request.
