# Supplementary material for: Unity Quantum Yield of High‐Entropy Quantum Dots Composited With Photonic Crystals for Information Encryption
Source: Adv Sci (Weinh). 2026 May 8;13(43):e75603. doi: 10.1002/advs.75603 (PMC13335938; doi:10.1002/advs.75603)
Supplement: Supplementary file 1 — Supporting File: advs75603‐sup‐0001‐SuppMat.docx. [file ADVS-13-e75603-s001.docx]

**Supporting Information**

**Unity Quantum Yield of High-Entropy Quantum Dots Composited with Photonic Crystals for Information Encryption**

*Maoyuan Huang, Ziqiang Tian, Binkun Xie, Haiyang Li, Bo Tan, Chang He, Shiliang Me**i*,* *Wanlu Zhang, Changchun Wang*, Ruiqian Guo**

M. Huang, B. Xie, H. Li, B. Tan, C. He, S. Mei, W. Zhang and R. Guo

Institute for Electric Light Sources

College of Intelligent Robotics and Advanced Manufacturing

Fudan University

Shanghai 200433, China

E-mail: meishiliang@fudan.edu.cn; rqguo@fudan.edu.cn

Z. Tian and C. Wang

State Key Laboratory of Molecular Engineering of Polymers and Department of Macromolecular Science

Laboratory of Advanced Materials

Fudan University,

Shanghai 200433, China

E-mail: ccwang@fudan.edu.cn

R. Guo

Yiwu Research Institute of Fudan University

Chengbei Road, Yiwu, Zhejiang 322000, China

R. Guo

Zhongshan-Fudan Joint Innovation Center

Zhongshan 528437, China

* Corresponding authors

**Experimental Section**

**Materials**

Copper iodide (CuI, 99.99%), zinc iodide (ZnI_2_, 99.99%), chromium(III) acetylacetonate (Cr(acac)_3_, 99.99%), gallium acetylacetonate (Ga(acac)_3_, 99.99%), selenium powder (Se, 99.99%), sulfur powder (S, 99.99%), zinc stearate (Zn(St)_2_, Zn 10%–12%), manganese chloride (MnCl_2_, 98 %), oleylamine (OLA, 80–90%), 1-octadecene (ODE, 90%), and 1-dodecanethiol (DDT, 98%) were purchased from Aladdin. All chemicals were used directly without further purification.

Styrene (St) and ethyl acrylate (EA) were purchased from Sinopharm Chemical Reagent Co., Ltd. and purified twice using an alkaline alumina column to remove the polymerization inhibitor before use. Sodium persulfate (SPS), sodium dodecyl sulfate (SDS), acrylic acid (AA), potassium hydroxide (KOH), divinylbenzene (DVB), 2-hydroxy-2-methylpropiophenone were purchased from Aladdin for using without further purification. Dowfax 2A1 and CO436 surfactants were purchased from Dow Chemicals. Butanediol diacrylate (BDDA) was purchased from the Tokyo Chemical Industry. Polydimethylsiloxane (PDMS) and the corresponding cross-linker (Sylgard 184 elastomer kit) were provided by Dow Corning.

**Synthesis of ZnI_2_ Precursor**

2 mmol of ZnI_2_ was dispersed in 4 mL of ODE and 4 mL of OLA with continuous stirring at 120℃.

**Synthesis of Zn(St)_2_ Precursor**

2 mmol of Zn(St)_2_ was dispersed in 2 mL of ODE and 0.5 mL of DDT with continuous stirring at 150℃.

**Synthesis of CZCrGSe QDs**

For a typical synthesis of CZCrGSe QDs, 0.07 mmol CuI, 0.28 mmol ZnI_2_, 0.14 mmol Cr(acac)_3_, 0.28 mmol Ga(acac)_3_, 1.3 mmol Se, 5 mL ODE, 5 mL OLA, and 2 mL DDT were added to a 50 mL three-necked flask and mixed. The mixture was degassed under a nitrogen atmosphere at room temperature for 30 min. After the system was fully purged with nitrogen, it was slowly heated from room temperature to 220℃ within 15 min and maintained for 5 min. The resulting colloidal solution was cooled to room temperature to obtain CZCrGSe core QDs. For purification, the crude solution was mixed with n-hexane and centrifuged at 7000 r/min for 3 min. The supernatant was collected, mixed with ethanol, and centrifuged at 8000 r/min for 3 min. The precipitate was collected and dispersed in n-hexane for further characterization.

**Synthesis of CZCrGSe/ZnSe QDs**

After the formation of CZCrGSe QDs, the mixture was heated from 220℃ to 240℃ within 1 min. The ZnI_2_ precursor was slowly injected into the reaction system in batches (2 mL each, with 10 min intervals). The resulting colloidal solution was cooled to room temperature to obtain CZCrGSe/ZnSe QDs. The final product was purified as described above.

**Synthesis of CZCrGSe/ZnSe/ZnS QDs**

After the formation of CZCrGSe/ZnSe QDs, the Zn(St)_2_ precursor solution was slowly injected into the reaction system at 240℃ and maintained for 60 min. The resulting colloidal solution was cooled to room temperature to obtain CZCrGSe/ZnSe/ZnS QDs. The final product was purified as described above. All the corresponding parameters of different groups are listed in **Table S4**.

**Synthesis of CZMnCrGSeS/ZnSeS/ZnS QDs**

For a typical synthesis of CZMnCrGSe/ZnSe/ZnS QDs, the procedure was similar to that of CZCrGSe/ZnSe/ZnS QDs, except that 0.07 mmol CuI, 0.28 mmol ZnI_2_, 0.14 mmol MnCl_2_, 0.14 mmol Cr(acac)_3_, 0.28 mmol Ga(acac)_2_, 0.65 mmol Se, and 0.65 mmol S were weighed sequentially into the flask. Subsequent steps were identical to those for CZCrGSe/ZnSe/ZnS QDs. The final product was purified as described above.

**Synthesis of PS@P(EA-co-AA) Core-Shell Nanoparticles**

PS@P(EA-co-AA) core-shell nanoparticles were synthesized via a semi-continuous stepwise emulsion polymerization method. Specifically, 0.9 g styrene, 0.1 g BDDA, 0.06 g SDS, and 70 g deionized water were added to a 250 mL three-necked flask to form an emulsion. The emulsion was heated to 85℃, and 0.063 g SPS dissolved in 1.25 g water was added to initiate polystyrene seed emulsion polymerization. After 10 min of reaction, an emulsion containing 17.5 g styrene, 1.90 g BDDA, 22.5 g deionized water, 0.06 g SDS, 0.10 g KOH, 0.055 g Dowfax 2A1, and 0.05 g SPS was added dropwise to the flask at a flow rate of 0.5 mL/min to synthesize the polystyrene core. After further reaction for 30 min, another emulsion containing 1.80 g AA, 16.2 g EA, 28.5 g deionized water, 0.145 g Dowfax 2A1, 0.15 g KOH, 0.75 g CO436, and 0.13 g SPS was added dropwise at a flow rate of 0.7 mL/min to obtain PS@P(EA-co-AA) core-shell nanoparticles. The product was freeze-dried for 36 h and then ground for subsequent use. The particle size of PS@P(EA-co-AA) core-shell nanoparticles could be adjusted by changing the initial dosage of SDS.

**Preparation of Dual-Mode HE-QD@PC Films and Their Application in Information Encryption**

At room temperature, dual-mode high-entropy QD composited PC (HE-QD@PC) films were prepared via co-assembly of PS@P(EA-co-AA) core-shell nanoparticles and CZCrGSe/ZnSe/ZnS QDs using molecule-mediated shear-induced assembly technology (MSAT). Specifically, a mediator molecule solution was prepared by uniformly mixing 1 mL AA (mediator molecule), 200 μL DVB (cross-linking agent), and 70 μL 2-hydroxy-2-methylpropiophenone (photoinitiator) via shaking and ultrasonic treatment. Subsequently, 40 μL of the mediator molecule solution was mixed with a mixture containing 50 mg PS@P(EA-co-AA) core-shell nanoparticles and 3.8 mg CZCrGSe/ZnSe/ZnS QDs under vigorous stirring to form a viscous slurry. Then, 30 mg of the slurry was tightly sandwiched between two polyethylene terephthalate (PET) films by gentle manual pressing to form a PET-slurry-PET sandwich structure. The composite film was inserted into the gap between two inwardly rotating rollers of an open mill. The power was turned on to start the open mill, and the slurry was subjected to rotational extrusion and bending at room temperature. Under strong shear force, CZCrGSe/ZnSe/ZnS QDs were embedded into the soft P(EA-co-AA) shell, while PS@P(EA-co-AA) core-shell nanoparticles were driven to assemble into a periodic photonic structure. After repeated operations, the initial slurry was transformed into a dual-mode HE-QD@PC film. Finally, the resulting dual-mode HE-QD@PC film was cured via photopolymerization for 5 min. Dual-mode HE-QD@PC films with different colors (structural color and fluorescent color) were cut into pentagram patterns of the same shape and size. These pentagram patterns were carefully arranged and encapsulated into PDMS, followed by thermal curing to obtain a visual information encryption system.

**Characterization**

The absorption spectra of QDs were measured using an ultraviolet-visible spectrophotometer (F795S, Shanghai Lengguang). Photoluminescence (PL) and photoluminescence excitation (PLE) spectra were recorded using a fluorescence spectrophotometer (F97XP, Shanghai Lengguang). Photoluminescence quantum yield (PLQY) was measured using an Edinburgh FS5 fluorescence spectrometer equipped with an integrating sphere. X-ray diffraction (XRD) data were collected using a Rigaku SmartLab SE X-ray diffractometer at a scanning rate of 5°/min. Transmission electron microscopy (TEM) and high-resolution TEM (HRTEM) images were obtained using a JEOL JEM-2100F at an acceleration voltage of 200 kV, with an attached Oxford X-Max 80T energy-dispersive spectroscopy (EDS) detector for elemental content and distribution analysis. X-ray photoelectron spectroscopy (XPS) data were acquired using a Thermo Scientific ESCALAB 250Xi, with the C 1s peak calibrated at 284.8 eV. Time-resolved photoluminescence (TRPL) curves were measured using an Edinburgh FLS1000 steady-state/transient fluorescence spectrometer.





**Figure S1.** Schematic diagram of the preparation route of CZCrGSe/ZnSe/ZnS QDs.


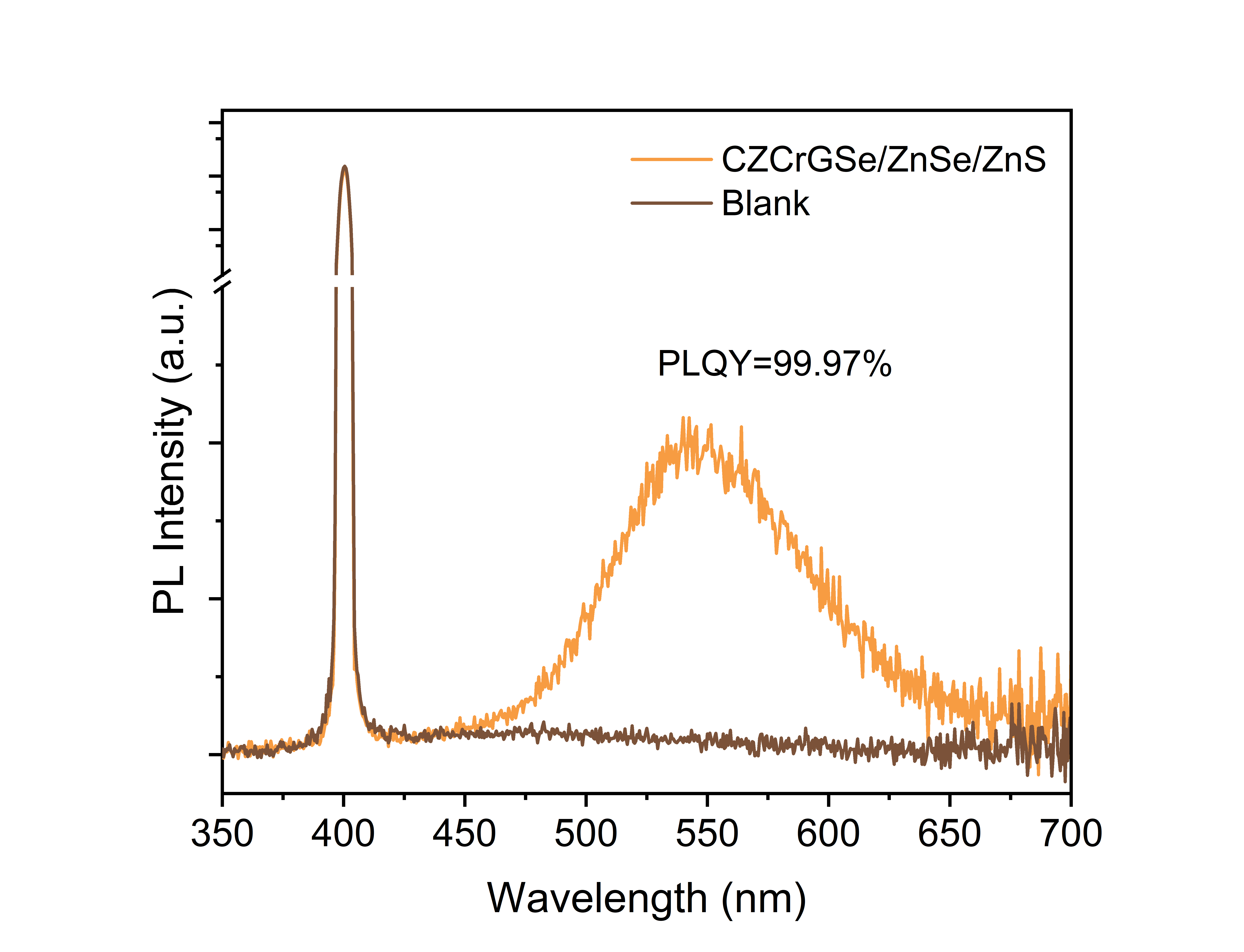


**Figure S2.** Absolute PLQY measurement of CZCrGSe/ZnSe/ZnS QDs with unity quantum yield.





**Figure S3.** TEM images of (a) CZCrGSe QDs, (b) CZCrGSe/ZnSe QDs and (c) CZCrGSe/ZnSe/ZnS QDs.





**Figure S4.** HRTEM images of (a) CZCrGSe QDs, (b) CZCrGSe/ZnSe QDs and (c) CZCrGSe/ZnSe/ZnS QDs.





**Figure S5.** HAADF-STEM image and the elementary mapping of CZCrGSe QDs.





**Figure S6.** HAADF-STEM image and the elementary mapping of CZCrGSe/ZnSe QDs.





**Figure S7.** EDS spectra of (a) CZCrGSe QDs, (b) CZCrGSe/ZnSe QDs and (c) CZCrGSe/ZnSe/ZnS QDs.





**Figure S8.** PLE spectra of CZCrGSe/ZnSe/ZnS QDs synthesized with different nucleation temperature.





**Figure S9.** (a) PL spectra and (b) absorption spectra of CZCrGSe/ZnSe/ZnS QDs synthesized with different shelling time. (c) PL spectra of CZCrGSe/ZnSe/ZnS QDs synthesized with different nucleation temperature and different shelling time.





**Figure S10.** PL spectra of CZCrGSe/ZnSe/ZnS QDs synthesized with different Cu:Ga ratio.





**Figure S11.** PL spectra of CZCrGSe_x_S_1-x_/ZnSeS/ZnS QDs synthesized with different Se:S ratio. Inset shows the photograph of CZCrGSe_x_S_1-x_/ZnSeS/ZnS QDs with different Se:S ratio under UV irradiation (*λ*=365 nm).


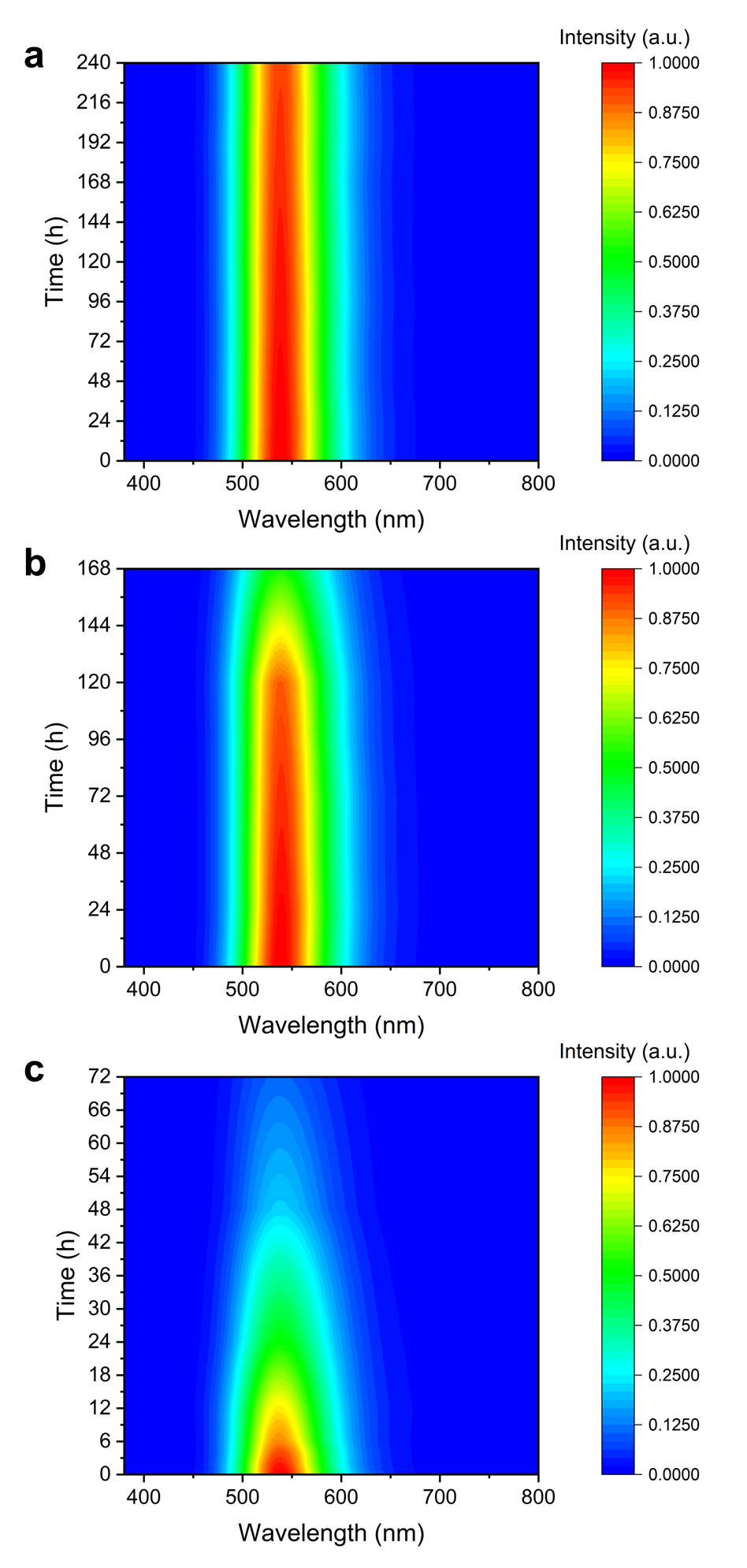


**Figure S12.** (a) Relative PL intensity of CZCrGSe/ZnSe/ZnS QDs under ambient environment. (b) Thermal stability of CZCrGSe/ZnSe/ZnS QDs at 80℃. (c) UV irradiation stability of CZCrGSe/ZnSe/ZnS QDs under the 365 nm UV light.


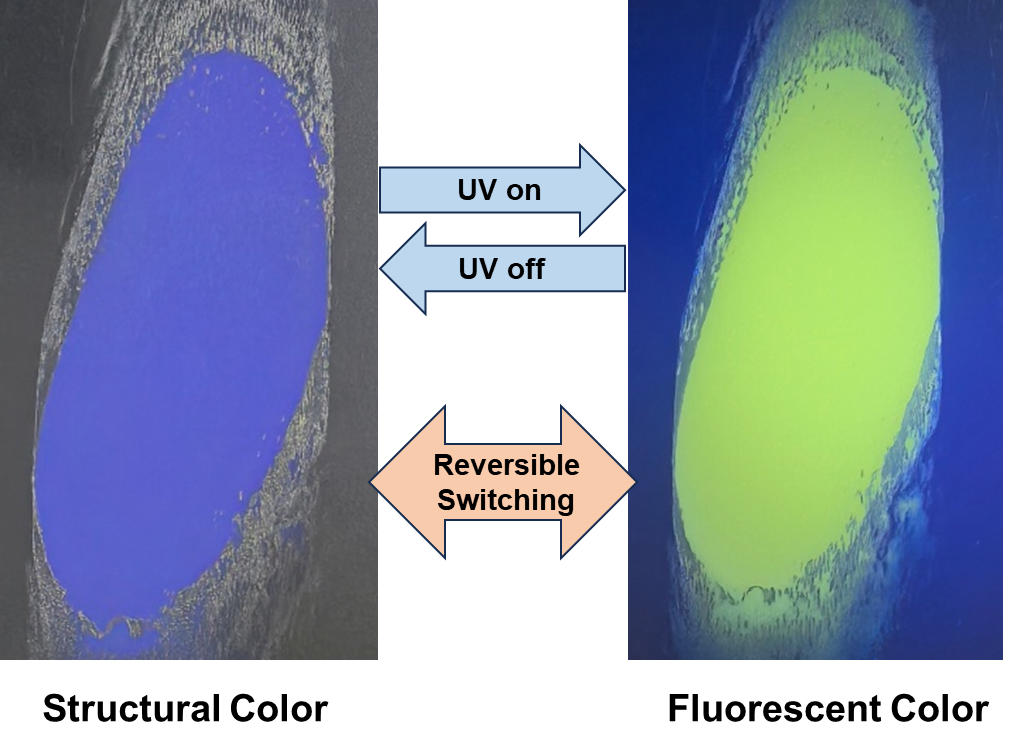


**Figure S13.** Photos of dual-mode HE-QD@PC films under daylight and UV irradiation (*λ*=365 nm).

**Table S1.** The comparison of previously reported alloy QDs with this work.

| **Years** | **QDs structure** | **PLQY**  **(%)** | **PL**  **(nm)** | **Ref.** |
| --- | --- | --- | --- | --- |
| 2014 | CuInS/ZnS | 80 | 578 | ^[1]^ |
| 2018 | CuGaSe/ZnSe | 77.73 | 573 | ^[2]^ |
| 2019 | ZnCuGaS/ZnS | 80 | 475 | ^[3]^ |
| 2021 | AgCuGaSe/ZnSe | 71.9 | 560 | ^[4]^ |
| 2022 | AgGaZnS | 16.7 | 470 | ^[5]^ |
| 2023 | AgInGaS/ZnS | 79.4 | 603 | ^[6]^ |
| 2023 | CuZnGaSeS | 72.2 | 543 | ^[7]^ |
| 2023 | ZnAgInGaS | 86.2 | 628 | ^[8]^ |
| 2024 | AgInGaS/AgGaS | 95 | 528 | ^[9]^ |
| 2024 | CuGaZnS | 90 | 478 | ^[10]^ |
| 2024 | CuInS/ZnS | 92.1 | 945 | ^[11]^ |
| 2024 | AgGaS/ZnS/ZnS | 96.4 | 508 | ^[12]^ |
| 2024 | CuInSe/ZnSe | 92.8 | 737 | ^[13]^ |
| 2024 | AgInGaZnS | 45 | 525 | ^[14]^ |
| 2025 | CuInZnS | 35 | 675 | ^[15]^ |
| 2025 | CuZnCrGaSe/ZnSe/ZnS | 100 | 540 | This work |

**Table S2.** Fitted parameters for PL decay curves of CZCrGSeS/ZnSeS/ZnS QDs and CZMnCrGSeS/ZnSeS/ZnS QDs synthesized with Cu:Mn=1:2 (intrinsic peak).

| **Cu:Mn** | ***A*_1_(%)** | ***τ*_1_ (μs)** | ***A*_2_ (%)** | ***τ*_2_ (μs)** | ***τ*_ave_ (μs)** |
| --- | --- | --- | --- | --- | --- |
| 1:0 | 56.86 | 0.1959 | 43.14 | 0.5795 | 0.46 |
| 1:2 | 48.97 | 2.62 | 51.03 | 26.06 | 24 |

**Table S3.** Fitted parameters for PL decay curves of CZMnCrGSeS/ZnSeS/ZnS QDs synthesized with Cu:Mn=1:2 and Cu:Mn=1:8 (Mn^2+^ peak).

| **Cu:Mn** | ***A*_1_(%)** | ***τ*_1_ (μs)** | ***A*_2_ (%)** | ***τ*_2_ (μs)** | ***A*_3_ (%)** | ***τ*_3_ (****μs)** | ***τ*_ave_ (μs)** |
| --- | --- | --- | --- | --- | --- | --- | --- |
| 1:2 | 20.11 | 3.31 | 28.58 | 23.55 | 51.31 | 319.16 | 306.31 |
| 1:8 | 18.61 | 56.97 | 41.48 | 269.54 | 39.91 | 824.77 | 669.27 |

**Table S4.** Main parameters of the reaction of high-entropy QDs.

| **Main parameters** | **Nucleation**  **temperatures** | **Molar ratios of Cu/Cr** | **Molar ratios of Cu/Ga** | **Molar ratios of Se/S** | **Molar ratios of Cu/Mn** |
| --- | --- | --- | --- | --- | --- |
| **Group 1** | **X_1_** | 1:2 | 1:4 | 10:0 | 1:0 |
| **Group 2** | 220℃ | **X_2_** | 1:4 | 10:0 | 1:0 |
| **Group 3** | 220℃ | 1:2 | **X_3_** | 10:0 | 1:0 |
| **Group 4** | 220℃ | 1:2 | 1:4 | **X_4_** | 1:0 |
| **Group 5** | 220℃ | 1:2 | 1:4 | 5:5 | **X_5_** |

**Group 1** (Nucleation temperatures: **X_1_** = 180℃, 200℃, 220℃ and 240℃)

**Group 2** (Molar ratios of Cu/Cr: **X_2_** = 1:0, 1:1, 1:2, 1:4 and 1:8)

**Group 3** (Molar ratios of Cu/Ga: **X_3_** = 1:1, 1:2, 1:4, 1:8 and 1:16)

**Group 4** (Molar ratios of Se/S: **X_4_** = 10:0, 7:3, 5:5, 3:7, 1:9 and 0:10)

**Group 5** (Molar ratios of Cu/Mn: **X_5_** = 1:0, 1:1, 1:2, 1:4 and 1:8)

**References**

[1] J.-H. Kim, K.-H. Lee, D.-Y. Jo, Y. Lee, J. Y. Hwang, H. Yang, *Applied Physics Letters* **2014**, *105*, 133104.

[2] S. Mei, G. Zhang, W. Yang, X. Wei, W. Zhang, J. Zhu, R. Guo, *Applied Surface Science* **2018**, *456*, 876-881.

[3] S.-Y. Yoon, J.-H. Kim, K.-H. Kim, C.-Y. Han, J.-H. Jo, D.-Y. Jo, S. Hong, J. Y. Hwang, Y. R. Do, H. Yang, *Nano Energy* **2019**, *63*, 103869.

[4] J. Wei, Z. Hu, W. Zhou, Y. Qiu, H. Dai, Y. Chen, Z. Cui, S. Liu, H. He, W. Zhang, F. Xie, R. Guo, *Journal of Colloid and Interface Science* **2021**, *602*, 307-315.

[5] X. Xie, J. Zhao, O. Lin, Z. Yin, X. Li, Y. Zhang, A. Tang, *The Journal of Physical Chemistry Letters* **2022**, *13*, 11857-11863.

[6] Z. Hu, H. Lu, W. Zhou, J. Wei, H. Dai, H. Liu, Z. Xiong, F. Xie, W. Zhang, R. Guo, *Journal of Materials Science & Technology* **2023**, *134*, 189-196.

[7] Z. Fu, H. Lu, H. Liu, Y. Xing, Z. Piao, H. Dai, W. Zhang, R. Guo, *Applied Materials Today* **2023**, *34*, 101922.

[8] S. Zhang, L. Yang, G. Liu, S. Zhang, Q. Shan, H. Zeng, *ACS Applied Materials & Interfaces* **2023**, *15*, 50254-50264.

[9] Y. Kim, A. Imran Channa, Y. Lee, Y. Kong, H.-M. Kim, Y.-H. Kim, S. Min Park, D. Kim, H. Yang, *Chemical Engineering Journal* **2024**, *486*, 150219.

[10] D. Hou, P. Lv, W. Niu, Z. Guan, L. Wang, J. Zhao, X. Li, H. Ye, A. Tang, *The Journal of Physical Chemistry Letters* **2024**, *15*, 7516-7523.

[11] Z. Liu, C. Hao, Y. Sun, J. Wang, L. Dube, M. Chen, W. Dang, J. Hu, X. Li, O. Chen, *Nano Letters* **2024**, *24*, 5342-5350.

[12] H. X. Lu, H. Liu, Z. Z. Fu, Y. Y. Chen, H. Q. Dai, Z. Hu, W. L. Zhang, R. Q. Guo, *Journal of Materials Science & Technology* **2024**, *169*, 235-242.

[13] W. Lian, D. Tu, X. Weng, K. Yang, F. Li, D. Huang, H. Zhu, Z. Xie, X. Chen, *Advanced Materials* **2024**, *36*, 2311011.

[14] X. Xie, J. Zhao, O. Lin, W. Niu, Y. Li, L. Wang, L. Li, Z. Yin, X. Li, Y. Zhang, A. Tang, *Nano Letters* **2024**, *24*, 9683-9690.

[15] H. Chen, H. Li, B. Dong, L. Hou, D. Yu, W. Wang, X. Xie, C. de Mello Donega, L. Cao, C. Xia, *Chemical Engineering Journal* **2025**, *516*, 164062.
